# Key Sources of Information and Support for Adults With Coeliac Disease: Coeliac Associations, Dietitians, Social Media and Cookbooks

**DOI:** 10.1111/jhn.70202

**Published:** 2026-01-19

**Authors:** Yvonne Jeanes, Humayun Muhammad, Ruksar Nishat, Katie Kennedy, Cristian Costas‐Batlle, Nick Trott, Sue Reeves

**Affiliations:** ^1^ School of Life and Health Sciences University of Roehampton London UK; ^2^ Dr Schär UK Ltd Birchwood Park Warrington UK; ^3^ Nutrition and Dietetics Department Bradford Teaching Hospitals NHS Foundation Trust Bradford UK; ^4^ Academic Unit of Gastroenterology Sheffield Teaching Hospitals Sheffield UK

**Keywords:** coeliac, dietitian, digital, gluten, social media

## Abstract

**Introduction:**

There is variable and often inadequate access to dietitians with expertise in coeliac disease (CeD) and limited data on where patients access support for their only treatment: adhering to a gluten‐free diet. This study aimed to provide an up‐to‐date report of where adults with CeD source useful information.

**Methods:**

A cross‐sectional online survey was undertaken during 2024. The survey was designed by specialist dietitians and a gastroenterologist. Questions addressed diagnosis, demographics, sources of gluten‑free diet information, their perceived usefulness, and exposure to inaccurate information. Adults with CeD were recruited through a customer database of GF foods in the UK and via social media.

**Results:**

Data from 675 adults with CeD showed that a high proportion rated information from coeliac associations (74%), peers with CeD (61%), cookbooks (56%), dietitians (50%), and the internet (52%) as highly useful, whereas only 15% reported information from General Practitioners (GPs) as highly useful. Among participants who regularly used social media (*n* = 360), 87% reported feeling connected to the CeD community; primary motivations for using social media were seeking peer support, gluten‑free recipes, information on new foods, dining options, and travel. However, nearly half (46%) indicated that they had received or accessed incorrect information via social media. Only 20% reported following a dietitian on social media.

**Conclusion:**

Coeliac associations remain a highly valued source of information and support. Social media is also widely used by adults with CeD, highlighting the need for greater dietitian engagement on these platforms as part of patient education and ongoing support.

## Summary

- Resources from Coeliac UK, a patient association, were considered highly useful and reliable.
- Only 15% of participants reported information from General Practitioners (GPs) to be highly useful, compared with 50% for dietitians.
- Gluten‐free cookbooks and online recipes were a particularly useful resource, alongside hearing from other people living with CeD.
- Social media users found social media very useful, though they reported a high proportion of incorrect information.

## Graphical abstract

Six hundred and seventy‐five adults with coeliac disease completed an online survey. Coeliac UK, dietitians, cookbooks and online recipes and other people with coeliac disease were considered extremely/very useful by the majority. Only 15% reported GPs to be extremely/very useful. Participants reported that social media was very useful, however, it also included incorrect information.

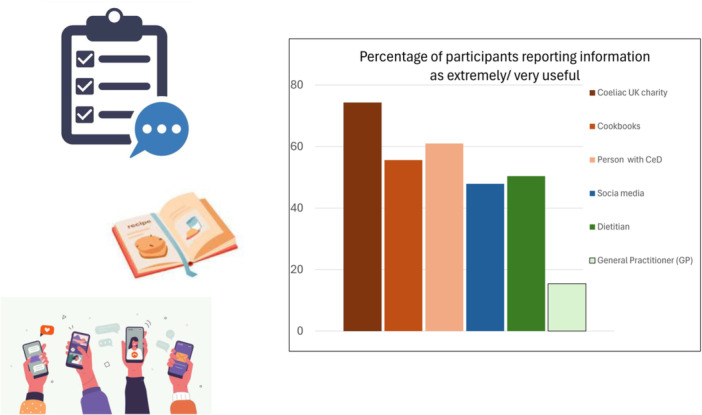

## 1 Introduction

The global prevalence of coeliac disease (CeD) is estimated at 1.4% [1]. CeD develops in response to little‐known environmental factors in genetically susceptible individuals [2]; it is a multi‐system autoimmune disorder characterised by a permanent loss of tolerance to dietary gluten, which leads to intestinal inflammation in these individuals. Gluten is a protein composite found in wheat, barley and rye; grains ubiquitously found in breads, pasta, pastries and cakes. Dietary adherence to a gluten‐free (GF) diet is paramount, as this is the only treatment available for CeD. Duodenal histological improvement, after removal of gluten from the diet, reverses the malabsorption state related to CeD and reduces CeD‐related morbidity [3, 4].

Adherence to a GF diet is widely accepted as challenging; it requires the individual to gain knowledge and modify behaviours; external barriers include a lack of widespread availability and high cost of GF foods [5, 6]. Additionally, foods, which are naturally GF, may come in contact with gluten during their harvesting, preparation, cooking and serving processes. The highly restrictive nature of the GF diet imposes a significant burden on patients, which can negatively impact their daily lives [7, 8]. Following the diagnosis of a chronic disease, patients commonly undergo an adjustment period as they learn to manage and live with the condition.

Limited education about the disease and limited practical guidance for following the GF diet is an attributing factor to inadequate adherence [9]. Having access to relevant and reliable information is important for educating and aiding new patients in disease management. Many individuals with a chronic disease turn to digital content for education about their condition and treatment [10], and the digital space enables patients to connect with each other forming communities and sharing their experiences.

Healthcare provision at diagnosis and follow‐up care is highly variable [11]. Recent studies from the US and UK have highlighted a high proportion of adults with CeD cannot access follow up care [12, 13]. Regular follow‐up with a healthcare professional is recommended within international guidelines [14, 15, 16], and patients with a lower health literacy and lower GF dietary knowledge highly valued healthcare reviews [12]. Individuals can find themselves with limited insight to their condition due to inadequate access to healthcare professionals with expertise in CeD and the GF diet [17].

The quality of freely accessed digital content on CeD is extremely variable; McNally et al. reviewed CeD content from 98 websites in 2012, reporting that many websites were not sufficiently accurate, comprehensive nor transparent to be considered sufficiently trustworthy and reliable for patients [18]. Data from social media platforms reported that the majority of CeD content was personal narratives posted by self‐promotors, parents of children with CeD, or those with a commercial interest, whereas there were only a few medical providers posting content [19, 20].

Individuals with CeD often experience feelings of being different and excluded [17]; thus, there is a role for healthcare professionals to promote ongoing support beyond appointments. Rej et al. evaluated group‐based healthcare, which enabled greater peer support [21]. Social media enables sharing of emotional and practical support through personal narratives [22]; this predominantly non‐healthcare professional content offers a different perspective, however, can present challenges in terms of accuracy of information [23, 24].

Considering the shift towards social media use, this study aimed to provide an up‐to‐date report of where UK adults with CeD source useful information.

## 2 Methods

A cross‐sectional online survey was designed to collate information on sources of information for CeD and living GF, as well as their perceived usefulness. The survey was designed with input from gastroenterology specialist dietitians. Questions included diagnosis, demographics, information sources for GF diet and their usefulness plus whether incorrect information was accessed/received (full survey in Supporting Information). To collect the broadest scope of information sources, we had an open‐ended question: *“If you have found a source of information particularly useful, please give us a short description.”* This information was collated through familiarisation, coding and generating themes for the most common information sources. Inclusion criteria required participants to be > 18 years, have a diagnosis of CeD and sufficient comprehension of the English language to complete the questionnaire. A prior study reporting sources of information was relatively small [25] (*n* = 82), thus we aimed to recruit over 300 participants. During 2024, using online survey software (Jisc), the survey link was promoted to a customer database of a manufacturer of GF foods in the United Kingdom and via social media. It is unknown how many potential participants received the survey link, and thus, the survey response rate could not be calculated. Descriptive chi‐squared data analysis was undertaken within the SPSS statistical package, version 28 (IBM Corp.). Presentation of the study aligns with STROBE guidance for observational studies. Ethical approval was granted through the procedures of the University of Roehampton Ethics Committee.

## 3 Results

Questionnaire data from 675 adults with coeliac disease were included for analysis (82% female, 95% white, 98% living in the UK, as shown in Table 1). Only 6% were 18–25 years old, 49% were 26–55 years and 45% were > 55years old. Half (*n* = 344; 51%) were current members of Coeliac UK, a charity which provides advice, support and research funding, offering some resources free of charge and more comprehensive support through a low‐cost subscription. The majority (84%) had been diagnosed for > 1 year, and 88% reported experiencing symptoms on ingestion of gluten.

**TABLE 1:** Descriptives of study population.

	Males	Females	Total *n* = (%)
Total (*n* = 1 prefer not to say)	117 (17.3)	556 (82.4)	675
Age categories*: 18–25	5 (4.3)	36 (6.5)	41 (6.1)
26–55	51(43.6)	282 (50.6)	334 (49.4)
> 55 years	61 (52.2)	234 (42.1)	295 (43.7)
Ethnicity: White	111 (94.9)	529 (95.1)	641 (95)
Asian	3 (2.6)	18 (3.2)	21 (3.1)
Length of time since diagnosis: < 1 yr	20 (17.1)	88 (15.8)	108 (16)
1–5 yr	29 (24.8)	142 (25.5)	172 (25.5)
3–10 yr	26 (22.2)	112 (20.1)	138 (20.4)
Over 10 years	42 (35.9)	214 (38.5)	257 38.1)
Symptoms: Yes	98 (83.8)	495 (89.2)	595 (88.1)
Following GF diet: Strict GF diet	67 (57.3)	387 (69.6)	456 (67.6)
Trying GF, not always sure	12 (10.3)	30 (5.4)	42 (6.2)
Usually GF some/rare intentional	26 (22.2)	115 (21.6)	141 (20.8)
GF most of the time	10 (8.5)	24 (4.3)	34 (5.0)
No restrictions	2 (1.7)	0 (0.0)	2 (0.3)
Confidence in managing GF diet: Very	53 (45.3)	284 (51.1)	337 (49.9)
Fairly	51 (43.6)	239 (43.0)	291 (43.1)
Neither	11 (9.4)	20 (3.6)	31 (4.6)
Not confident	2 (1.7)	13 (2.3)	15 (2.2)
Not at all confident	0 (0.0)	0 (0.0)	0 (0.0)
Current member of Coeliac UK: Yes	45 (38.5)	298 (53.6)	344 (51)
No, though was previously	26 (22.2)	109 (19.6)	136 (20.1)
No	46 (39.3)	149 (26.8)	195 (28.9)

* 5 missing data.

The majority (68%) indicated that they were following a strict GF diet, 20% indicated ‘usually GF with some/rare gluten ingestion’, 6% ‘were trying to follow a GF diet but not always sure’ and 5% ‘were GF most of the time’ (Table 1). Seventy percent of participants who are members of Coeliac UK and 58% who were not member reported to be following a strict GF diet (*p* < 0.01). Half of the participants (50%) indicated they were very confident in managing their coeliac disease with a GF diet; 81% of these had indicated they were following a strict GF diet.

**FIGURE 1:**
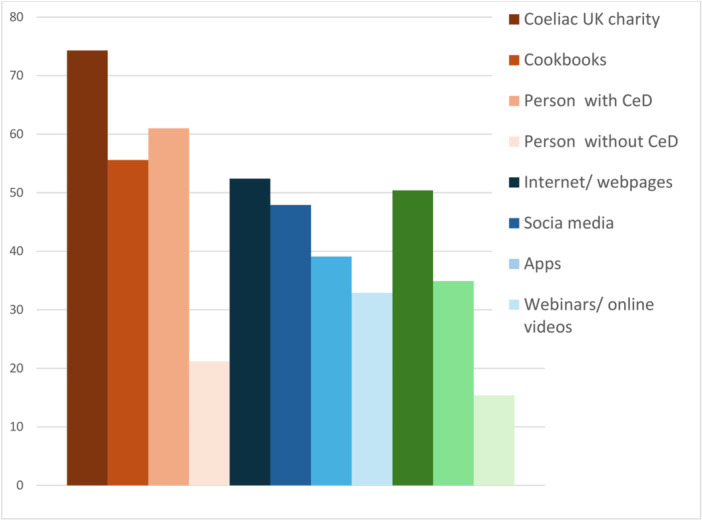
Gluten‐free diet information sources reported to be extremely/very useful by adults with CeD.

From nine predetermined information sources, Coeliac UK was rated as extremely/very useful by the highest percentage of participants and consistent across all age groups (Tables 2 and 3). Members of Coeliac UK patient association highly rated their resources, with 85% rating them as ‘extremely/very useful’ for information received about GF diets (Table 1). Even 61% of non/past members of Coeliac UK rated the Coeliac UK charity resources as ‘extremely/very useful’ (Table 2, Figure 1). Nearly two‐thirds of participants (61%) rated the information from another person with CeD as ‘extremely/very useful’, whereas information from someone without CeD was predominately considered not useful (Table 2). Over 50% considered cookbooks and the internet/web pages to be ‘extremely/very useful’ (Figure 1). Overall, 48% of participants reported that social media was extremely/very useful. In a subgroup analysis of the 130 participants who used social media once or more per day to seek information and support related to coeliac disease, 70% reported that it was extremely/very useful. Fewer participants in the older age categories rated social media as useful compared with younger participants (Table 3). Of note, a larger proportion of older participants indicated that they rarely/never used social media (56–65 yrs: 59.7%, > 66 years: 69.9%) compared with younger participants (18–25 yrs: 26.8%, 26–35 yrs: 32.2%).

**TABLE 2:** Reported usefulness of information received about gluten‐free diet by adults with coeliac disease (CeD).

Information source	*N* = *	% Extremely/very useful	% Somewhat useful	% Not/a little bit useful
Coeliac UK charity (all)	628	74.3	17.0	8.7
‐ Non/past members	286	60.9	24.5	14.6
‐ Current members	342	85.4	10.8	3.8
Person with CeD	540	61.0	25.6	13.4
Person without CeD	537	21.2	16.4	62.4
Cookbooks	585	55.6	28.9	15.5
Newspapers/magazines	541	15.5	28.1	56.4
Internet/webpages	643	52.4	33.4	14.2
Webinars/online videos	365	32.9	31.5	35.6
Socia media	545	47.9	25.1	27.0
Apps	462	39.1	29.9	31.0
Healthcare professionals				
Dietitian	575	50.4	19.7	29.9
Gastroenterologist	596	34.9	25.2	39.9
General Practitioner (GP)/Family doctor	633	15.4	21.2	63.4

* Missing data/participants response *I do not know/not sure* are not included.

**TABLE 3:** Percentage of responses reporting ‘extremely/very useful’ of information received about gluten free diet by adults with coeliac disease (CeD) split by age.

Information source	18–25	26–35	36–45	46–55	56–65	> 66 yrs	*p* value
	*N* = 41	*N* = 90	*N* = 117	*N* = 127	*N* = 137	*N* = 156	
Coeliac UK charity	69.3	74.1	75.2	74.4	71.7	77.4	0.90
Person with CeD	73.6	57.5	72.6	69.6	60.4	43.8	< 0.01
Person without CeD	35.3	16.0	21.3	25.0	17.3	21.3	0.47
Cookbooks	55.6	54.6	58.2	60.1	52.0	54.7	0.47
Newspapers/magazines	15.1	14.9	10.3	20.2	14.1	16.8	0.08
Internet/webpages	60.9	60.7	62.3	58.7	46.6	38.4	< 0.01
Webinars/online videos	50.0	40.0	37.7	33.3	23.8	26.8	0.02
Socia media	70.0	56.5	54.7	60.4	32.7	27.9	< 0.01
Apps	42.4	39.4	39.7	48.3	43.0	27.2	0.07
Healthcare professionals							
Dietitian	43.2	53.6	43.3	47.6	53.5	55.3	0.40
Gastroenterologist	34.3	32.1	30.7	34.5	30.7	45.0	0.51
General Practitioner (GP)/Family doctor	15.4	8.0	12.5	15.3	12.3	26.0	0.09

Only 15% of participants rated General Practitioners/Family doctors as ‘extremely/very useful’ for information received about GF diets, whereas 50% of participants considered information from dietitians as ‘extremely/very useful’ and a further 20% as ‘somewhat’ useful (Table 2).

In response to asking if there was any particular information source that was particularly useful, 291 participants responded, most popular themes included ‘Other people with coeliac disease/online community’, ‘Social media/blogs’, ‘Coeliac UK charity’ and ‘GF recipes – in books and online’ (Table 4). Additionally, responses highlighted that participants needed to find information due to receiving inadequate support from healthcare professionals when diagnosed with CeD:

‘*I have found the online community to be extremely useful when I received very little information initially from healthcare professionals’.*

‘*I wish Instagram had been around when I was first diagnosed as I was given very limited support by the NHS (National Health Service)’.*

‘*My GP (General Practitioner) just said I had coeliac disease so stop eating gluten and look at Coeliac UK’.*

‘*I didn't get any help after being diagnosed, just a DEXA (Dual‐energy X‐ray absorptiometry) scan. No dietitian, no follow ups so learnt everything from there (social media)’.*

**TABLE 4:** Most popular responses to ‘*was any information source particularly useful?’* with corresponding participant quotes.

Other people with coeliac disease/online community	Social media/blogs
‘*Meeting people online with the same condition made me feel confident and assured that I'm not alone’.* ‘*Best support I have received is from speaking to others living with coeliac disease’* ‘*Tend to find advise from others that have coeliac to be most helpful’* ‘*Coeliac disease for beginners on Facebook. It's been a life saver to talk to people in my position with advice that comes from experience’.* ‘*I've got a lot of info and support chatting to other shoppers in the ‘free from aisle in the supermarket!’* ‘*Both my dad and my uncle are coeliac so that has been my main source of information’*	‘*Blogs and reviews of places to eat are my best source of truth when going out or on holiday’* ‘*Gluten free influencers in social media, they help giving recipes, restaurants or coffee shops they went where they take cross contamination seriously, etc’.* ‘*TikTok accounts useful as they have improved my confidence in going out for meals’* ‘*Influencers ‐ coeliac nutritionists and Instagram chefs’* ‘*Social media ‐ following other coeliacs’* ‘*Social media posts where people tell you of different places in specific parts of the UK/the world for coeliacs to eat gf’.*
Coeliac association (Coeliac UK)	GF recipes – in books and online
‘*Being able to get information from trusted places online such as Coeliac UK is brilliant, especially when I was first diagnosed and had a lot of questions’.* ‘*Coeliac UK is the only reliable source’* ‘*The Coeliac UK app for scanning barcodes is absolutely essential to me’.*	‘*Buying cookery books have helped me have a varied diet’* *‘XX cookbooks are fantastic, all GF and easy to follow recipes’* *Instagram recipes!* *Recipes ‐ online and books XX cookbooks and blog for recipes*

*XX: removed identification of specific author/influencer.

Of the 360 participants who used social media at least once a week: 85% engaged with Facebook, 59% Instagram, 36% YouTube, 24% TikTok, 18% X, 13% LinkedIn and 5% Threads. Of these participants (80% female, 97% white), 8% were 18–25 years old, 63% were 26–55 years and 29% were > 55 years old; a younger demographic to the total study population. Nineteen percent of participants (*n* = 130) used social media at least once a day, 34% one to several times a week, and 47% rarely or never to seek information and support related to CeD.

**FIGURE 2:**
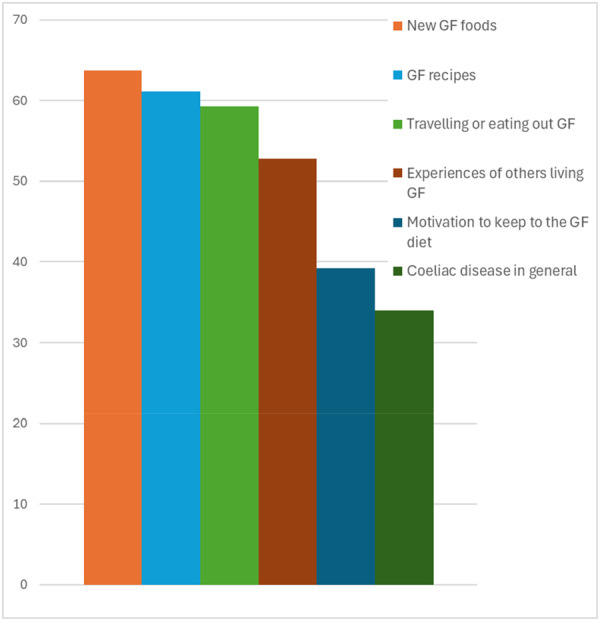
Percentage of participants indicating topics on social media as ‘extremely/very useful’ (*n* = 360).

Participants who regularly used social media (at least once a week: *n* = 360), 19% indicated they felt very connected with the CeD community, 68% felt somewhat connected and 13% were not connected/not interested. Regular users of social media, 42% felt very confident in managing their CeD with a GF diet. Only 12% reported following a doctor on social media and 20% were following a dietitian on social media. Figure 2 highlights the proportion of participants indicating topics on social media as ‘extremely/very useful’, these correspond with the responses to the open‐ended question on reasons for social media use in relation to coeliac disease and gluten‐free living which revealed two main purposes: obtaining information (e.g., gluten‐free recipes, new foods, dining out, and travelling) and accessing support from others with coeliac disease:

‘*To check foods to see if it's edible, recipes, not to feel isolated’.*

‘*To ask questions and find others in the community, and to find recommendations for places to get gf food’.*

‘*Up to date information’*

Participants who regularly use social media, 28% reported they had made dietary changes based on information found on social media, examples given were predominately positive:

‘*Ingredient changes in foods or recalled foods.*’

‘*I have had coeliac for 30 years and didn't know soy sauce had gluten in until a blogger posted it on instagram!’*

‘*Learnt how to actually make different types of bread at home due to the cost of gluten free products in supermarkets. Learnt about new products so I can make dishes I used to be able to eat ….’*

‘*Cut out a risky may contain and added in items I hadn't realised were safe’*

**FIGURE 3:**
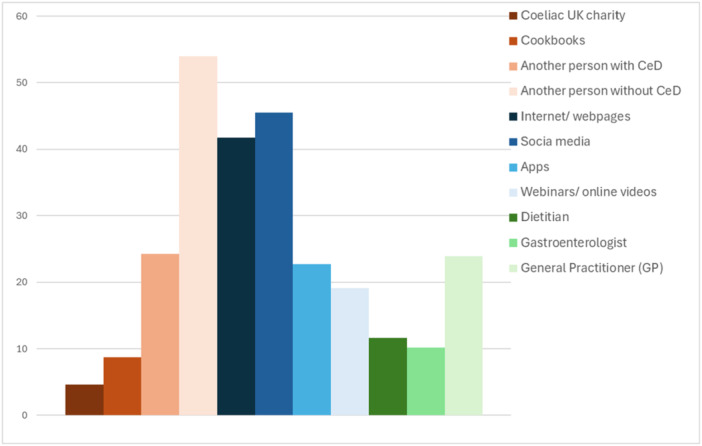
Proportion of UK adults with coeliac disease who reported having received/accessed incorrect information from differing sources.

As shown in Figure 3, participants most frequently identified incorrect information as originating from individuals without coeliac disease (54%), social media (46%), and internet/webpages (42%). In contrast, the sources least associated with incorrect information were Coeliac UK, cookbooks, dietitians, and gastroenterologists.

## 4 Discussion

Our study reports a large proportion of adults with CeD consider information from Coeliac UK patient association, another person with CeD, cookbooks, dietitians and the internet as highly useful. Participants who engaged regularly with social media also reported that they felt connected with the CeD community. They valued social media for the support offered by others with CeD, access to GF recipes, improved awareness of new GF foods, and advice on dining out and travelling. Social media can be regarded as an important resource for facilitating adherence to a GF diet. However, a high proportion of participants had accessed incorrect information in the online and social media spaces and only 20% were following a dietitian on social media. Awareness of diverse information sources and patient support networks among healthcare professionals may contribute to improved patient care, particularly in the context of the limited availability of healthcare professionals with comprehensive knowledge of CeD [12].

Adherence to a GF diet is challenging, within our cohort 68% reported strict adherence to a GF diet, this is a similar proportion to other studies [4, 26]. Half of our cohort were members of Coeliac UK, with a higher proportion than non‐members following a strict GF diet; studies have consistently reported membership with a coeliac association is positively associated with GF dietary adherence [25, 26, 27]. Coeliac support groups offer practical advice and support to patients with CeD, and healthcare professionals are advised to signpost patients to Coeliac UK within the UK guidelines [14]. Eighty‐five percent of the participants who were members of Coeliac UK indicated their resources were extremely/very useful. A Spanish study reported that the local patient associations were the preferred choice of information if they had a query about the GF diet [10], and a UK study indicated members of Coeliac UK experienced a lower dietary burden when following the GF diet [12]. Factors influencing dietary adherence are multifactorial; adherence is not easy to achieve due to the ubiquitous presence of gluten in staple foods, gluten cross‐contact, inadequate food labelling, limited availability of GF meals and the social pressures to name a few [4, 9, 26]. It is important for healthcare professionals to be aware of the value patients place on coeliac associations, such as Coeliac UK, and the wider social support to enable better long‐term care.

The study highlights how recipes, whether online or in cookbooks have an enduring usefulness. Over 10 years ago, Silvester et al. reported cookbooks as one of the most used sources of information in a Canadian study [25]. The GF diet excludes so many foods, and replicating the organoleptic properties of gluten in GF baked goods is challenging, thus the creative solutions to replicate gluten appears to be highly valued as indicted in the current study.

Over half of participants reported dietitians provided ‘extremely/very useful’ information, and studies have shown specialist dietitians with expertise in CeD positively impact patient outcomes, for example, through identifying involuntary gluten ingestion [28], improving nutritional adequacy and reducing the dietary burden [29]. However, it is concerning to note 30% of participants considered dietitians to be only a little bit/not useful, this could be due to limited access to dietitians with expertise in CeD [12, 30] and requires further exploration. Patients who have access to healthcare professionals place a high value on them having expertise in CeD; a UK survey reported 62% of adults with CeD would like to have an appointment with a dietitian, with 80% of those requesting a specialist dietitian [12]. An online dietitian‐led prerecorded webinar is an initiative that provides instant access to dietetic expertise [31]. General Practitioners (GPs) by their very nature are generalist; the majority of our cohort indicated they were not considered useful sources of information, this is concerning as in the UK many adults with CeD are managed within primary care. Studies in CeD are limited, though within IBS management, dietary information from GPs was perceived as trustworthy but simplistic with the familial and social impacts not being addressed [32]. Training on CeD and the broad range of factors influencing GF dietary adherence is very important, not only for patients, but also for GPs. Dietitians with expertise in CeD have a key role in providing accessible information to those with varying health literacy, with a focus on easing the dietary burden associated with living GF [29].

Information from a person without CeD was considered to be of little benefit, and over half of the participants reported receiving incorrect information from people without CeD. However, a person with CeD living GF was reported to be highly useful source of information and support; we did not distinguish how that information was acquired, it could have been in person, via online blogs or social media interactions. Our findings support previous studies whereby patients reported the benefits of interactions with other people with CeD [17, 25, 33]. A Spanish study reported adults with CeD preferred source of information included the internet, where they included social networks, with 18% of participants reporting useful guidance from specific blogs or influencers, and 12% from peers inclusive of online community groups [10]. However, a Canadian study of adolescents reported that whilst 51% of those surveyed visited a social media site at least daily, they did not use social media as an information source for GF living [33].

Many adults with CeD connect through social media to exchange their experiences living GF and health information, with over half of our participants using social media to seek information and support related to CeD at least once a week. In a subgroup of participants who used social media once or more per day, 70% reported that it was extremely/very useful, perceive usefulness reduced in older age categories, which also included less people using social media. This agrees with a study of 221 adults recruited from a Saudi Arabia coeliac support group; 96% used social media platforms to help manage their disease, of these 94% agreed that social media was helpful in increasing their adherence with the GF diet [23]. In our study, half of regular social media users found it to be extremely/very useful for hearing about experiences of others living GF and learning about new GF foods, GF recipes, travelling and eating out GF. These aspects are highly practical elements for navigating through the real world and preventing inadvertent gluten ingestion. Many of our cohort highlighted the importance of community and connecting with others with CeD. Social media allows people to create these digital communities to feel seen and supported. Healthcare professionals could signpost patients to social media for these aspects of support. To understand the role of social media in GF dietary adherence and its value in different sub‐populations, a robustly designed study is needed.

However, while some online information and support networks are valued, people need to have good health literacy skills to understand and use information in ways that promote and maintain good health. It is important that patients are made aware that not all sources are accurate or evidence‐based. Social media is a double‐edged sword, which is reflected in our data; whilst 47% find it extremely/very useful for living GF, 45% also reported it as a source of incorrect information. There is scope for a much greater presence of dietitians and doctors on social media; in our study, only 12% reported following a doctor and 20% following a dietitian on social media. Patients are using social media to support the required self‐management of CeD; thus, should we as healthcare professionals be thinking of ways of incorporating it into our practice to better support patients?

Whilst our study offers several insights, it needs to be recognised that our cohort is likely to have a bias towards adults who are more digitally literature, due to our recruitment methods via email and social media. Thus, our study may be overestimating the perceived usefulness of social media, as we have a high proportion using social media regularly. Postal surveys have shown to recruit a less digitally literature patient group and would offer broader insight, a recent study reported participants who completed the online survey had better access to good quality internet and were more confident in online technology, compared with those who completed the paper survey [34]. Additionally, we had a predominance of female participants and white ethnicity, thus limiting the generalisability of findings. Targeted recruitment to ethnic minorities and the survey being available in languages other than English would have improved the study. We have presented patient‐reported data on their interpretation of misinformation as per the questionnaire text, without providing users with a definition such as the Cambridge dictionary: *‘false information, given either by mistake or deliberately’* [35], thus it is difficult to interpret what patients understood as misinformation. A strength of the study was recruiting participants outside of the healthcare setting and coeliac association, with the aim of reporting from a real‐world situation with varied healthcare support.

Coeliac associations continue to provide a highly valued information and support service for adults with CeD; this is particularly important as many patients do not have access to regular healthcare reviews. The usefulness of information from healthcare professionals was highly variable and highlights the need for patients to be offered support from someone with expertise in CeD to enable an impactful interaction with the healthcare team.

Recognising the limited healthcare support available for adults with CeD, there is a clear role for the internet and social media in providing valuable information that reduces the GF dietary burden, supports eating out, increases awareness of GF foods and provides a growing CeD online community that many participants value. There is a clear need for a greater presence of dietitians and doctors within these online spaces as part of healthcare provision for patients. We recommend that healthcare professionals maintain awareness of high‐quality social media and online resources to support patients in accessing the CeD community and obtaining ongoing, practical information for living gluten‐free.

## Author Contributions

Yvonne M. Jeanes, Humayun Muhammad, Ruksar Nishat and Sue Reeves designed the study. Ruksar Nishat, Katie Kennedy, Cristian Costas‐Batlle and Nick Trott were involved in recruitment. Yvonne M. Jeanes, Humayun Muhammad and Ruksar Nishat analysed the data. Yvonne M. Jeanes drafted the manuscript. All authors provided critical feedback and contributed to the final version of the manuscript. All authors approved the final version of the manuscript, and it has not been published elsewhere.

## Funding

The authors received no specific funding for this work.

## Ethics Statement

Ethical approval was granted by the procedures of the University of Roehampton Ethics Committee.

## Conflicts of Interest

Katie Kennedy is employed by a GF food manufacturer, and Yvonne Jeanes received funding from a GF food manufacturer to present the findings at a conference. The GF food manufacturer had no input in the design, analysis nor reporting of the study findings.

## Transparency Declaration

The lead author affirms that this manuscript is an honest, accurate and transparent account of the study being reported. The reporting of this work is compliant with STROBE guidelines. The lead author affirms that no important aspects of the study have been omitted and that any discrepancies from the study as planned have been explained.

## Supporting information

Supplementary information blinded.

## Data Availability

The data that support the findings of this study are available from the corresponding author upon reasonable request.
