# Supplementary material for: Key Sources of Information and Support for Adults With Coeliac Disease: Coeliac Associations, Dietitians, Social Media and Cookbooks
Source: J Hum Nutr Diet. 2026 Jan 19;39(1):e70202. doi: 10.1111/jhn.70202 (PMC12816437; doi:10.1111/jhn.70202)
Supplement: Supplementary file 1 — Supplementary information blinded. [file JHN-39-0-s001.pdf]

Finding useful information for living gluten free

We invite you to take part in a short online survey. Your answers will provide valuable insights to inform better support for individuals with coeliac disease.

Study title: Where do adults living with coeliac disease find useful information for living gluten free?

Introduction: There is an enormous amount of information online, in books or from healthcare professionals, but knowing what is useful for helping you to live gluten free is valuable. The growth of social media has changed where many people gather and share information.

This study will investigate the usefulness of different sources of information and your confidence in the accuracy of the information

This study aims to find out where adults living with coeliac disease source useful information to help them live gluten free.

Participant Involvement: You will be asked questions about where you get information on living gluten free and how useful you find it. This survey takes approximately 10 minutes.

Benefits of Participation: As an adult living with coeliac disease, you will be able to share your experiences and insights contributing to a better understanding of the broader support systems for individuals with coeliac disease.

There is also an opportunity to be included in a prize draw for a £50 shopping voucher.

Confidentiality: All the details will be kept confidential. The information of identity will be coded and only the research team will have the rights to view the individual data. No identifiable data related to the participants will be used in reports and publications.

Investigators contact details:

Name: [redacted]

Name: [redacted]

Supervisors [redacted]

Informed consent statement:

I agree to take part in this research and am aware that I am free to withdraw at any point without giving a reason by contacting either [redacted] or [redacted] on given details. I understand that if I do withdraw, my data may not be erased but will only be used in an anonymised form as part of an aggregated dataset. I understand that the personal data collected from me during the course of the project will be used for the purposes outlined above in the public interest. I have been informed about and understand the University's Data Privacy Notice for Research Participants.

The information you have provided will be treated in confidence by the researcher and your identity will be protected in the publication of any findings. The purpose of the research may change over time, and your data may be re-used for research projects by the University in the future. If this is the case, you will normally be provided with additional information about the new project. Please note: if you have a concern about any aspect of your participation or any other queries, please raise this with the Supervisors. However, if you would like to contact an independent party, please contact the Dean of School.

Supervisor contact details: [redacted]

[redacted]

[redacted]

1. I hereby confirm that I have read and agreed to the participant consent form, I have been diagnosed with coeliac disease, I am over the age of 18years and I agree to take part in the study *

- ◯ Yes
- ◯ No

Background information

This information is required to demonstrate the survey has reached a representative sample of adults living with coeliac disease.

2. Please indicate your age: *

- ◯ 18-25 years
- ◯ 26-35 years
- ◯ 36-45 years
- ◯ 46-55 years
- ◯ 56-65 years
- ◯ 66 years and above
- ◯ prefer not to say

3. Please indicate your sex? *

- ◯ Male
- ◯ Female
- ◯ Other
- ◯ Prefer not to say

4. Choose one option that best describes your ethnic group or background *

- ◯ White
- ◯ Black/African/Carribean/Black British
- ◯ Asian/Asian British
- ◯ Mixed/Multiple Ethnic groups
- ◯ Other ethnic group
- ◯ Prefer not to say

5. Where do you live? *

- ◯ UK
- ◯ Europe, not UK

- North America
- South America
- Asia
- Australisa
- Africa
- Prefer not to say

Living gluten free

This information helps us to understand how you are living gluten free.

6. How long have you been diagnosed with coeliac disease? *

- ◯ Less than 1 year
- ◯ 1-5 years
- ◯ 3-10 years
- ◯ More than 10 years

7. Do you experience symptoms if you eat gluten? *

- ◯ Yes
- ◯ No
- ◯ Do not know

8. Are you a member of Coeliac UK? *

- ◯ Yes
- ◯ No
- ◯ No, though I have been previously

9. Please describe your *current* diet *

- ◯ No restrictions (eating foods that contain gluten)
- ◯ Gluten-free diet most of the time
- ◯ Usually gluten-free with rare intentional gluten consumption
- ◯ Usually gluten-free with rare unintentional gluten consumption
- ◯ Trying to follow a gluten-free diet but not always sure
- ◯ Strict gluten-free diet

10. How <u>confident</u> are you in managing your coeliac disease with a gluten free diet?

- ◯ Very confident

- Fairly confident
- Neither confident or unconfident
- Not confident
- Not at all confident

Places you find information on living gluten free

11. Please rate the usefulness of any information you have received **about gluten-free diets** from each of the following sources. *

GP/ Family doctor

- ☐ Not useful
- ☐ A little bit useful
- ☐ Somewhat useful
- ☐ Very useful
- ☐ Extremely useful
- ☐ I do not know/ not sure

Hospital Doctor (Gastroenterologist)

- ☐ Not useful
- ☐ A little bit useful
- ☐ Somewhat useful
- ☐ Very useful
- ☐ Extremely useful
- ☐ I do not know/ not sure

Dietitian

- ☐ Not useful
- ☐ A little bit useful
- ☐ Somewhat useful
- ☐ Very useful
- ☐ Extremely useful
- ☐ I do not know/ not sure

Nutritionist

- ☐ Not useful
- ☐ A little bit useful
- ☐ Somewhat useful
- ☐ Very useful
- ☐ Extremely useful
- ☐ I do not know/ not sure

Other healthcare professional (Nurse/Pharmacist)

- [ ] Not useful
- [ ] A little bit useful
- [ ] Somewhat useful
- [ ] Very useful
- [ ] Extremely useful
- [ ] I do not know/ not sure

Coeliac UK, any type of information, online, booklets, Apps

- [ ] Not useful
- [ ] A little bit useful
- [ ] Somewhat useful
- [ ] Very useful
- [ ] Extremely useful
- [ ] I do not know/ not sure

Another person with Coeliac disease

- [ ] Not useful
- [ ] A little bit useful
- [ ] Somewhat useful
- [ ] Very useful
- [ ] Extremely useful
- [ ] I do not know/ not sure

Family or friends without Coeliac disease

- [ ] Not useful
- [ ] A little bit useful
- [ ] Somewhat useful
- [ ] Very useful
- [ ] Extremely useful
- [ ] I do not know/ not sure

Newspapers/Magazines

- [ ] Not useful
- [ ] A little bit useful
- [ ] Somewhat useful
- [ ] Very useful
- [ ] Extremely useful
- [ ] I do not know/ not sure

Internet/ Webpages

- ☐ Not useful
- ☐ A little bit useful
- ☐ Somewhat useful
- ☐ Very useful
- ☐ Extremely useful
- ☐ I do not know/ not sure

Webinars/ online videos

- ☐ Not useful
- ☐ A little bit useful
- ☐ Somewhat useful
- ☐ Very useful
- ☐ Extremely useful
- ☐ I do not know/ not sure

Social Media

- ☐ Not useful
- ☐ A little bit useful
- ☐ Somewhat useful
- ☐ Very useful
- ☐ Extremely useful
- ☐ I do not know/ not sure

Apps

- ☐ Not useful
- ☐ A little bit useful
- ☐ Somewhat useful
- ☐ Very useful
- ☐ Extremely useful
- ☐ I do not know/ not sure

Cookbooks

- ☐ Not useful
- ☐ A little bit useful
- ☐ Somewhat useful
- ☐ Very useful
- ☐ Extremely useful
- ☐ I do not know/ not sure

Other

- ☐ Not useful
- ☐ A little bit useful
- ☐ Somewhat useful
- ☐ Very useful
- ☐ Extremely useful
- ☐ I do not know/ not sure

12. If you have found a source of information particularly useful please give a short description below:

13. Have your ever had misinformation/ incorrect information, about coeliac disease or living gluten free, from any of these sources? *

GP/ Family doctor

- ◯ Yes
- ◯ No
- ◯ Unsure

Hosptial doctor/ Gastroenterologist

- ◯ Yes
- ◯ No
- ◯ Unsure

Dietitian

- ◯ Yes
- ◯ No
- ◯ Unsure

Other healthcare professional (Nurse, pharmacist)

- ◯ Yes
- ◯ No
- ◯ Unsure

Coeliac UK

- ◯ Yes
- ◯ No
- ◯ Unsure

Another person with coeliac disease

- ◯ Yes
- ◯ No
- ◯ Unsure

Friends or family without coeliac disease

- ◯ Yes
- ◯ No

Unsure

Newspaper/ magazine

- Yes
- No
- Unsure

Internet/ webpages

- Yes
- No
- Unsure

Webinars/ online videos

- Yes
- No
- Unsure

Social Media

- Yes
- No
- Unsure

Apps

- Yes
- No
- Unsure

Cookobooks

- Yes
- No
- Unsure

Your use of Social Media & chance to opt into prize draw

14. What social media platforms do you regularly use? Tick all that apply *

- ☐ Facebook
- ☐ Instagram
- ☐ YouTube
- ☐ X (Twitter)
- ☐ LinkedIn
- ☐ TikTok
- ☐ Threads
- ☐ None

15. How often do you use social media to seek information and support related to Coeliac disease? *

- ◯ Many times, each day
- ◯ Once a day
- ◯ Several times a week
- ◯ Once a week
- ◯ Rarely
- ◯ Never

16. What is the main reason for using social media, related to coeliac disease/ living gluten free?

17. If you use social media, please rate the usefulness of social media on providing information on the following:

Coeliac disease in general

- [ ] Not useful
- [ ] A little bit useful
- [ ] Useful
- [ ] Very useful
- [ ] Extremely useful

Travelling or eating out gluten-free

- [ ] Not useful
- [ ] A little bit useful
- [ ] Useful
- [ ] Very useful
- [ ] Extremely useful

Experiences of others living gluten-free

- [ ] Not useful
- [ ] A little bit useful
- [ ] Useful
- [ ] Very useful
- [ ] Extremely useful

Gluten-free recipes

- [ ] Not useful
- [ ] A little bit useful
- [ ] Useful
- [ ] Very useful
- [ ] Extremely useful

New gluten-free foods

- [ ] Not useful
- [ ] A little bit useful
- [ ] Useful
- [ ] Very useful
- [ ] Extremely useful

Motivating you to keep to the gluten-free diet

- [ ] Not useful
- [ ] A little bit useful
- [ ] Useful
- [ ] Very useful
- [ ] Extremely useful

18. Have you ever made dietary changes based on the information found on social media? *

- ○ Yes
- ○ No

19. If yes, please give an example of what dietary change you made

20. Do you follow any healthcare professionals on social media?

- [ ] Doctor
- [ ] Dietitian
- [ ] Not sure
- [ ] None

21. If you use social media, is there someone who posts particularly useful/ relevant content for you? Please indicate who they are

22. How connected do you feel to the coeliac disease community on social media? *

- ○ Not connected, not interested
- ○ Somewhat connected
- ○ Very connected, has importance to me

Thank you for taking the time to complete this survey

23. If you would like to be included in the prize draw for a £50 shopping voucher please provide your email address:

    Your email address will only be used to contact you if you have won the shopping voucher, and will be deleted two weeks after the survey closes.

24. If you have any other comments you would like to add, please do so here:

Adults with coeliac disease may benefit from probiotics, and particularly their cholesterol lowering impact. [redacted] is inviting adults with coeliac disease who are interested in knowing their cholesterol level and would consider joining a study to see if a probiotic supplement can help them. For more information click here.
